# Integrative transcriptomic, proteomic, and phosphoproteomic analysis on the defense response to *Magnaporthe oryzae* reveals different expression patterns at the molecular level of durably resistant rice cultivar Mowanggu

**DOI:** 10.3389/fpls.2023.1212510

**Published:** 2023-07-13

**Authors:** Weiye Peng, Yunsheng Wang, Xuanning Zeng, Wei Li, Na Song, Jing Liu, Bing Wang, Liangying Dai

**Affiliations:** ^1^ College of Plant Protection, Hunan Agricultural University, Changsha, Hunan, China; ^2^ Hunan Provincial Key Laboratory for Biology and Control of Plant Diseases and Insect Pests, Hunan Agricultural University, Changsha, Hunan, China

**Keywords:** *Magnaporthe oryzae*, integrative analysis, transcriptomic, proteomic, phosphoproteomic

## Abstract

Rice blast, caused by *Magnaporthe oryzae* is one of the most destructive diseases of rice (*Oryza sativa* L.) in most rice-cultivated areas worldwide. Mowanggu (MWG) is a traditional landrace rice variety in Yunnan with broad-spectrum and durable blast resistance against rice blast fungus. However, the underlying disease-resistance mechanisms remain unknown. An integrative transcriptomic, proteomic, and phosphoproteomic analysis of MWG was performed after inoculation with *M. oryzae* in this study. The transcriptomic and proteomic results revealed that MWG was moderately correlated at the transcriptional and protein levels. Differentially expressed genes and proteins were up-regulated and significantly enriched in protein phosphorylation, peroxisome, plant-pathogen interactions, phenylpropanoid metabolism and phenylalanine biosynthesis pathways. The phosphoproteomic profile and phosphorylated-protein-interaction network revealed that the altered phosphoproteins were primarily associated with reactive oxygen species (ROS), glycolysis, MAPK signaling pathways, and amino acid biosynthesis. In addition, a series of physiological and biochemical parameters, including ROS, soluble sugars, soluble protein and callus accumulation and defense-related enzyme activities, were used to validate the possible blast resistance mechanisms of MWG. The integrative transcriptomic, proteomic, and phosphoproteomic analysis revealed the different expression patterns at the molecular level of the durably resistant rice cultivar MWG after inoculation with *M. oryzae*, which provides insight into the molecular mechanisms of rice blast resistance.

## Graphical Abstract

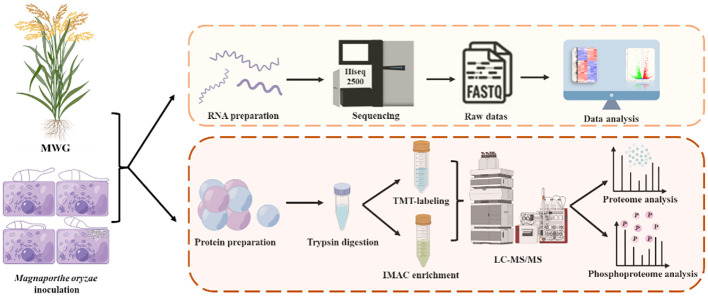

## 1 Introduction

Rice (*Oryza sativa*) is a cereal crop and a stable food for over half of the world’s population (Xu et al., 2020; Yu et al., 2021a). However, rice crops are regularly exposed to several biotic and abiotic stresses, with fungal diseases comprising one of the most serious threats to rice production (Li et al., 2020). Rice blast, caused by the ascomycete fungus *Magnaporthe oryzae*, reduces global rice yield by 10–30% annually with no effective treatment (Kim et al., 2020). An understanding of the molecular mechanisms of the rice–*M. oryzae* interaction could help to develop new blast control strategies.

The advent of RNA-sequencing (RNA-seq) and novel bioinformatics algorithms have enabled the rapid screening of potential hub genes and key pathways in plant–pathogen interactions (Fass et al., 2020). In the last decade, several transcriptomics studies have advanced our understanding of the interplay of rice-*M. oryzae*. In a previous transcriptome study on the resistance of rice IRBL18 mediated by blast resistance gene *Pi1* and IRBL22 mediated by *Pi9*, *OsWRKY* genes were identified as the main regulators of rice blast resistance (Qiu et al., 2020). Further expression patterns analyses revealed that OsWRKY47 was is significantly induced at 24 h post post-inoculation, and plants overexpressing OsWRKY47 exhibited enhanced are highly resistant to *M. oryzae* (Wei et al., 2013). In addition, RNA-seq was explored to study unique and significant differentially expressed loci and the sophisticated transcriptional reprogramming during immune responses in the broad spectrum Near Isogenic Lines (*PB1*, *Pi1*, *Pi9* and *Pi54*) at 24 hpi (Jain et al, 2017; Jain et al, 2019). In a similar RNA-seq analysis, several defense-related genes were dramatically up-regulated. Cellular basal metabolism and energy metabolism-related genes were significantly repressed in the resistant cultivar, GangYu129, harboring the durable blast resistance gene *Pi65* at 24 hpi (Wang et al., 2021).

However, several studies have shown that mRNA levels cannot accurately predict the abundance of the end protein products since the correlation between them is not linear (Ye et al., 2015; Atlasi et al., 2020). Besides, posttranslational modifications, protein-protein interactions and subcellular localization are often impossible to investigate through mRNA expression information. Thus, a comprehensive and quantitative proteomic study on RNA-seq is necessary to provide new insights into the molecular mechanisms of *M. oryzae* resistance. For example, 32 and 16 differentially expressed proteins are identified at 24 and 48 hpi using the proteomics approach in blast susceptible cultivar CO39. Two near-isogenic lines CN4a/4b, in response to the infection of pathogenic isolates with varying levels of pathogenicity, are also identified (Ma et al., 2019). This result suggests that host plants may have related but distinct defense mechanisms between different infection stages, and 24 hpi is the critical period of rice defense response against rice blast infection.

Recent studies have demonstrated that besides the abundance of the synthesized proteins, posttranslational modification (PTM) of the stress-related signaling proteins is particularly important to dynamically regulate signaling in response to invasion patterns originating from the pathogen (Liu et al., 2019). Protein phosphorylation is one of the most thoroughly studied PTM and has been estimated to affect about 30% of total protein in plants (Papamokos et al., 2021). For example, OsCERK1 functions in blast disease resistance by interacting with OsRLCK185 and transducing pathogen-associated molecular patterns immune signals into phosphorylation events to activate the MAPK Cascade (Wang C. et al., 2017). OsWRKY45 regulates the defense network mediated by salicylic acid signaling in rice, and its full activation requires phosphorylation (Thr266, Ser294, and Ser299) of OsMPK6 (Ueno et al., 2017). Nevertheless, the gel-based or mass spectrometry-based proteomics approach lacks the precision to detect low-abundant phosphoproteins (Farooq et al., 2022). Moreover, due to technical limitations, conventional experimental methods often only investigate the kinase-substrate pairs independently, making identifying modified sites on proteins difficult and cumbersome (Hou et al., 2015). Through new-generation high-throughput sequencing technologies of mass spectrometry and phosphopeptides enrichment, many studies have comprehensively identified phosphopeptides and phosphosites at the whole proteome level (Gao et al., 2020). A recent integrated proteomic and phosphoproteomic study characterized the changes in gene expression. Differentially phosphorylated proteins were found during pattern-triggered immunity (PTI) and effector-triggered immunity (ETI) in the tomato-*Pseudomonas syringae* pv. tomato (*pst*) pathosystem, revealing several potential ETI/PTI-specific markers and providing insight into the affected signaling pathways (Yu et al., 2021b).

Breeding rice varieties with durable blast resistance requires a deep understanding of the possible physiological and molecular mechanisms identified through extensive exploration and discovery of multi-omics techniques in rice. Upon entering the post-genome era, the emergence of next-generation sequencing has propped new -omics research. However, the integrative dynamics of the proteome and phosphoproteome in infected host plants and the resistance to *M. oryzae* in rice is unknown (Li and Xu, 2021). Multi-omics analyses on more rice varieties are required to provide information on the genetic basis of resistance. Herein, a rice-blast-resistant cultivar was selected as the research material. Mowanggu (MWG) is a japonica-type durable and broad-spectrum resistant rice cultivar cultivated for over 100 years in mountainous areas of Yunnan Province, China (Sun et al., 2022). Thus, differentially expressed genes and translated proteins are identified by integrating transcriptomic, proteomics, and phosphoproteomic analyses. Moreover, large-scale bioinformatics analyses, including pathway enrichment and phosphorylation motif and levels, were performed to assess the regulation networks of MWG against *M. oryzae*. This study contributes to the understanding of the regulatory mechanisms of rice-*M. oryzae* pathosystem, providing new potential strategies for improving rice disease resistance.

## 2 Materials and methods

### 2.1 Plant material and growth condition

Durably resistant rice cultivar MWG was used in this study. The seeds were surface-sterilized and germinated in distilled water for four days. The distilled water was changed daily. Budding seeds were selected and transferred to planting trays filled with sterile medium soil under standard physiological conditions of 16 h light/8 h dark at 26 ± 1°C with 80% humidity. Three-week-old healthy rice seedlings (three-leaf to four-leaf stage) were placed in an inoculation chamber and used for inoculation with highly virulent strain 110-2 of *M. oryzae*.

### 2.2 Fungal inoculation

Cultivation of the blast fungus *M. oryzae* strain 110-2 and fungal inoculations of rice plants were performed with few modifications to previously described methods (Peng et al., 2021). The *M. oryzae* strain 110-2 was grown on tomato-oat culture medium at 26 ± 2°C for two weeks. Conidia of *M. oryzae* were collected after rinsing with sterile water and filtering through two layers of gauze. The spore suspension was diluted to 5 × 10^5^/mL with 0.02% Tween-20. All the experimental groups were fine sprayed with the working spore suspension, whereas control plants were mock-inoculated with only 0.02% Tween-20 lacking *M. oryzae* spores. Post spray-inoculation, inoculated plants were incubated in a growth chamber at 28°C and over 80% relative humidity for 24 h. Each rice sample was a mixture of leaf tissue at 24 hpi and frozen in liquid nitrogen after collection. The remaining seedlings were maintained for an additional week for disease evaluation. Each experiment was performed using three independent biological and three technical replicates.

### 2.3 Experimental design

The multi-omic studies’ high quality, confidence, and coverage derive crucial information about plant-phytopathogen interaction. The underlying defense mechanism of the durably resistant rice cultivar (Mowanggu rice blast resistance) was further investigated. The strategy used to access the multi-omics of *M. oryzae*-inoculated plants is illustrated in the Graphical abstract (By Figdraw).

### 2.4 RNA isolation and library preparation for Illumina Hiseq 2500

Total RNA samples were extracted from leaf tissues as described in a previously study (Peng et al., 2022). RNA purity and concentration were examined using a Nanodrop-1000 spectrophotometer (Thermo Fisher Scientific, Waltham, MA, USA), and RNA quality was further evaluated using the Agilent 2100 bioanalyzer instrument (Agilent, La Jolla, CA). RNA‐integrity numbers (RIN) were obtained for each sample, and only those with an RIN factor above 8.5 were selected for preparing sequencing libraries. Libraries were pooled and sequenced with paired-end 100-bp reads using the ultrahigh-throughput Illumina HiSeq 2500 platform (Illumina, San Diego, California).

A functional characterization analysis was performed within each sample of differentially expressed genes (DEGs) using the agriGO v2.0 and the KEGG databases that explored the strengths of the software tools Goatools v0.5.7 and KOBAS v3.0 web server (Bu et al., 2021). The heatmap was constructed according to the absolute log_2_ fold change, using a comprehensive bioinformatic software package TBtools v1.098769 (Chen et al., 2020). The transcriptional data have been deposited in the *NCBI* Gene Expression Omnibus (GEO) and are accessible through GEO Series accession number. 

### 2.5 Protein extraction and digestion

Briefly, the samples were removed at −80 °C. An appropriate amount of leaf tissue was weighed, milled in a ball mill (45 Hz, 60 s, repeated twice), and pre-cooled in liquid nitrogen. Subsequently, the sample powder was gently resuspended in 1 ml of extraction buffer (150 mM NaCl, 100 mM sodium phosphate, 5 mM EDTA, 5 mM EGTA, 1% Triton X-100, 1 mM PMSF, 1x phosphatase inhibitors II & III, 1x protease inhibitor cocktail, and 50 μM Mg-132). Lysis was performed through sonication, and the lysate was centrifuged at 12,000 rpm for 10 min at 4°C. A 20% trichloroacetic acid solution was added to the supernatant, and the well-mixed solutions were left to stand at 4°C for 2h. After centrifugation, the supernatant was carefully removed, and the pellet was thoroughly washed thrice with pre-cooled acetone. The final pellet was dissolved in 8-M urea, and protein concentrations were measured using Easy Protein Quantitative Kit (BCA), according to the manufacturer’s protocol (Transgen, Beijing, China).

Samples were reduced with 5 mM of dithiothreitol (DTT) at 56°C for 40 min, and alkylated with 11 mM iodoacetamide for 20 min at ambient temperature in the dark. Finally, the resultant sample was diluted to a final urea/thiourea concentration below 2 M. The diluted protein samples were incubated with a 1/40 trypsin/protein ratio at 37°C overnight and a 1/40 trypsin/protein mass ratio for a second digestion for 4 h.

### 2.6 Tandem mass tag labeling and enrichment of phosphorylated peptides

Following enzymatic digestion, the tryptic peptide mixtures were desalted with solid-phase extraction cartridges (Strata X C18 SPE column; Phenomenex) and freeze-dried under a vacuum. The dried peptide mix was dissolved in 0.5-M TEAB labeled using a 6-plex Tandem Mass Tag (TMT) kit. The labeled peptide samples were resuspended in 500 μl loading buffer (1% glutamate, 2% trifluoroacetic acid (TFA), and 65% acetonitrile (ACN), and TiO2 beads were added into the solution (26°C, over 30 min). The centrifuged mixture was eluted twice with wash buffer A (0.5% TFA and 65% ACN) and wash buffer B (0.1% TFA and 65% ACN). Finally, the phosphopeptides were serially eluted in binding buffer A (50% ACN and 5% NH_4_OH) and B (60% ACN and 10% NH_4_OH). The resultant peptide mixtures were pooled, lyophilized, and stored at –20°C until use.

### 2.7 Liquid chromatography–mass spectrometry analysis

Two microliters (corresponding to 1 µg protein) of the peptide mixtures were loaded using an autosampler to a 75 μm × 500 mm EASY-Spray Pepmap C18 column packed with a 100 μm × 5 mm EASY-Spray Pepmap C18 trap column and linked to an autosampler EASY-nLC 1000 HPLC system (Thermo Fisher Scientific). The separated peptides were eluted using a solvent system containing (A) HPLC‐grade water with 0.1% formic acid and (B) ACN with 2% HPLC‐grade water and 0.1% formic acid at a flow rate of 200 ml/min over a 2-h gradient. Data-dependent acquisition was performed in a positive ion data-dependent mode using an Orbitrap Fusion Lumos Tribrid™ mass spectrometer at a nominal resolution of 120,000 (mass-to-charge ratio *m*/*z*=200) and full scan survey spectra between 400 and 1500 *m*/*z*. The resultant peptides were subjected to a nanospray ionization (NSI) source followed by tandem mass spectrometry (MS/MS) in Q Exactive™ Plus coupled online to the ultraperformance liquid chromatograph (UPLC). The electrospray voltage was set at 2.0 kV. The polypeptide was further selected for MS/MS using a normalized collision energy setting of 30%, and the ion fragments were measured using the Orbitrap at a resolution of 15,000. The automatic gain control target value was 50,000, and the constant first mass was 110 m/z.

### 2.8 Data analysis

LC-MS/MS-acquired data were extracted using Proteome Discoverer software version 2.4 (ThermoFisher Scientific). Saved spectra were collected iteratively using the Byonic search engines (Protein Metrics Inc. v4.0.2). The precursor mass tolerance was set at ± 10 ppm and fragment ion mass tolerance at ± 0.02 Da while choosing the digesting enzyme trypsin and permitting maximum two-missed tryptic cleavages. In the Mascot search, TMT-label (229.163) on lysine residue and peptide N-terminus and Cysteine carbamidomethylation (57.021) were configured as fixed modifications. The protein N-terminal acetylation (42.011), methionine oxidation (15.995), and serine/threonine/tyrosine phosphorylation (79.966) were defined using the software platform as variable modifications. Protein identification was performed using the universal protein knowledgebase Database (UniProt) with a false discovery rate of 0.01. Integration tolerance was performed at 20 ppm using the default centroid method for the reporter ion Quantifier node. Ion signal intensities correction was performed using isotopic impurities according to the IsoCor tool protocol. A minimal average reporter signal-to-noise ratio (S/N) with a threshold width of 2 and a co-segregate threshold with 100% efficiency was set for each subject. For channel quantitation, the S/N value for all peptides was summed across each TMT channel, normalized, and corrected such that each sample had an identical summed value. “Unique plus razor peptides” mode was used to identify and quantify peptides in groups.

Peptides of different abundance were extracted from the Byonic output list after a higher-energy collisional dissociation MS2 scan. Only peptides identified in at least two biological replicates were run for further analyses. Statistical analysis was performed using SPSS software (version 22.0, Chicago, IL, USA). Differences between the two groups were analyzed using a one-way analysis of variance or Students’ t-test. P-value ≤ 0.05 was considered statistically significant.

## 3 Results

### 3.1 Identification of the changes in the transcriptome profile of rice variety MWG in response to *Magnaporthe oryzae* infection

**Figure 1:**
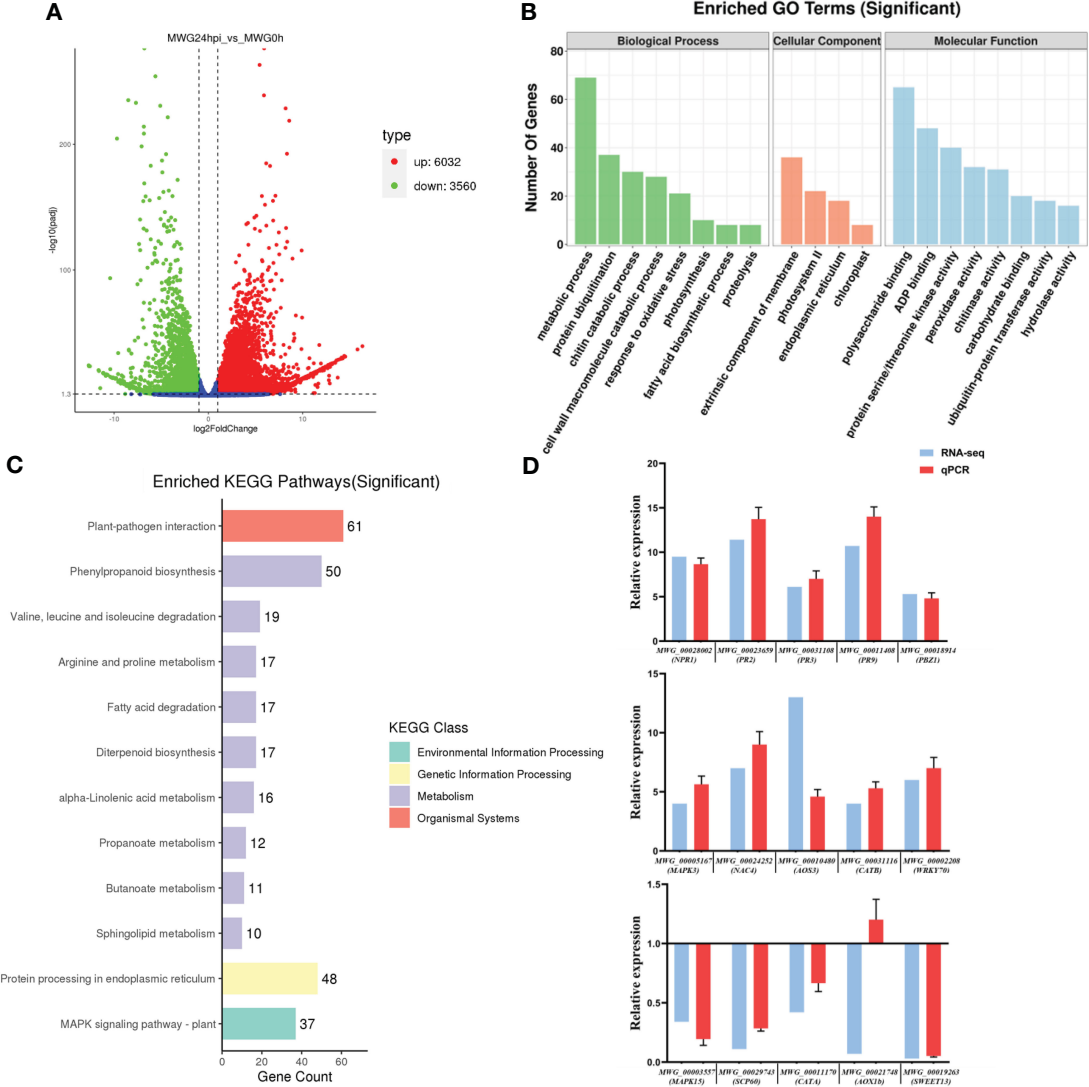
Transcriptome analysis of MWG in response to *Magnaporthe oryzae* infection. **(A)** Volcano plot for differential gene expression. **(B)** Go functional category analysis of the differentially expressed genes. **(C)** KEGG enrichment analysis of the differentially expressed genes. **(D)** Comparison of RNA-Seq and qPCR results of 15 genes. For data normalization, *OsUBQ* was used as a reference gene.

To investigate the differential transcriptional regulation of MWG in response to rice *M*. *oryzae* infection, MWG leaves were inoculated with a suspension of *M*. *oryzae*. RNA-seq analysis was performed on samples at 0 and 24 h after inoculation (hpi). Transcriptome results revealed that each library generated clean reads at 45.0 ± 3.5 million, and the total mapped rates were between 91.98% and 95.84% (**Supplementary Table S1**). Additionally, the average Q20 (sequencing error rate lower than 1%), Q30 (sequencing error rate lower than 0.1%), and GC content of the clean reads were 96.15%, 90.50%, and 56.40%, respectively. Principal component analysis (PCA) demonstrated a distinct separation between *M. oryzae* infected and control samples, indicating highly reproducible and reliable results of the overall experimental procedures (**Supplementary Figure S1**). Five main types of alternative splicing events were found. Among them, Alternative 3’ splice site had the highest number of splicing events (5858), and mutually exclusive exon had the least number (298) (**Supplementary Figure S2**). A total of 10852 differentially expressed genes (DEGs) were identified at 24 h post-inoculation, consisting of 4052 and 6800 genes which were up-regulated and down-regulated, respectively, with changes in expression levels greater than 1.5 or lower than 0.67 and a p-value less than 0.05. This result suggested that *M. oryzae* infection causes massive transcriptional reprogramming in MWG (**Figure 1A**).

AgriGO database search included GO terms, such as “membrane”, “photosystem I”, and “chloroplast”, on the cellular component level. The molecular function (MF) category mainly comprised DEGs involved in “polysaccharide binding”, “ADP binding”, “protein serine/threonine kinase activity”, “peroxidase activity”, and “chitinase activity”. In the biological process sub-categories involving “metabolic process”, “protein ubiquitination”, “chitin catabolic process”, “cell wall macromolecule catabolic process”, and “response to oxidative stress”, were the most highly represented groups (**Figure 1B**). After the KEGG pathway analysis, DEGs were involved in many processes, including most pathways associated with Plant-pathogen interaction, Phenylpropanoid biosynthesis, Protein processing in the endoplasmic reticulum, and MAPK signaling pathway (**Figure 1C**). RNA-seq analysis with 15 selected DEGs was confirmed using qPCR: pathogenesis-related (PR) gene (NPR1, PR2, PR3, PR9, PBZ1), abiotic stress responses-related (NAC4, WRKY70, SCP60, SWEET13), reactive oxygen species (ROS)-related gene (CATA, CATB), and hormone biosynthesis (AOS3, AOX1b). The expression patterns of MWG_00010480 (AOS3) showed a weak correlation compared with MWG_00021748 (AOX1b).^28,29^ However, the remaining genes displayed strong correlations, suggesting that the RNA-seq data were real and reliable (**Figure 1D**).

### 3.2 Proteomic profiling of rice variety MWG in response to *Magnaporthe oryzae* infection

**Figure 2:**
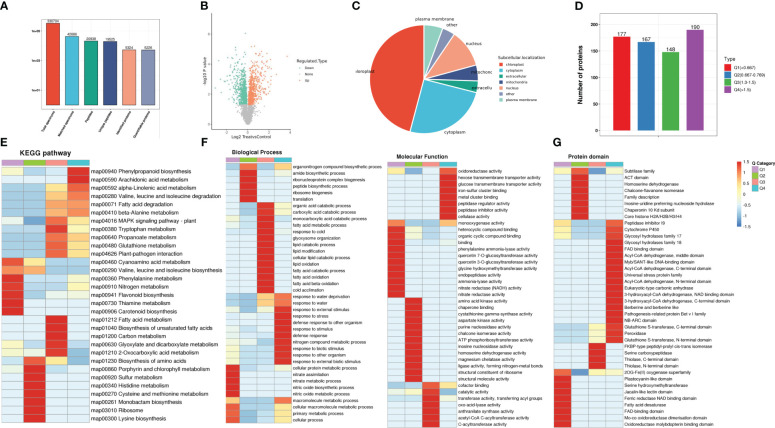
Proteome analysis of MWG in response to *Magnaporthe oryzae* infection. **(A)** Validation of proteins identified by proteomics. Volcano plot **(B)** and subcellular locations **(C)** of differentially expressed proteins. **(D)** Grouping the of differentially expressed protein. KEGG enrichment **(E)**, GO function enrichment **(F)** and protein domain analysis **(G)** of differentially expressed proteins.

This study identified changes in the proteome of MWG in response to M. oryzae infection using the TMT technique. A total of 42,686 matched spectra, 20,938 unique peptides 5226, and quantifiable proteins were identified in the proteomic analysis (**Figure 2A**). The quality control data indicated that the length of peptide segments and the theoretical molecular weight of protein are concentrated at 8-22 amino acids and 10-100 kDA, consistent with the LC-MS results (**Supplementary Figure S3**). Regarding the application of rigorous screening criteria (absolute fold change ≥1.5 and adjusted p-value ≤0.05), 338 up-regulated and 344 down-regulated proteins were identified (**Figure 2B**). Bioinformatics predictions of the subcellular localization of the DEPs were investigated, and the results revealed that DEPs are mainly localized in the chloroplast, cytoplasm, and nucleus. However, a few DEPs are localized in the plasma membrane or other parts (**Figure 2C**).

The DEPs were categorized into four groups according to their expression patterns, including Q1-Q4 (**Figure 2D**). The functional enrichment of the four groups was analyzed in three dimensions: KEGG pathway, GO annotation, and protein domain. For the KEGG pathway analysis, a significant enrichment of “plant-pathogen interaction” and “MAPK signaling pathway” in the Q3 and Q4 groups was identified (**Figure 2E**). GO enrichment analysis indicated that response-related processes (defense response and biotic stimulus-response) and molecular function (oxidoreductase and cellulase activity) were significantly enhanced in up-regulated proteins (Q4). In contrast, numerous metabolic processes (cellular protein, nitric oxide, and macromolecule metabolic) were negatively affected (**Figure 2F**). In the up-regulated proteins, several domains, such as NB-ARC, peroxidase, and glycosyl hydrolases, were highly enriched (**Figure 2G**).

### 3.3 Integration of transcriptomic and proteomic Data

**Figure 3:**
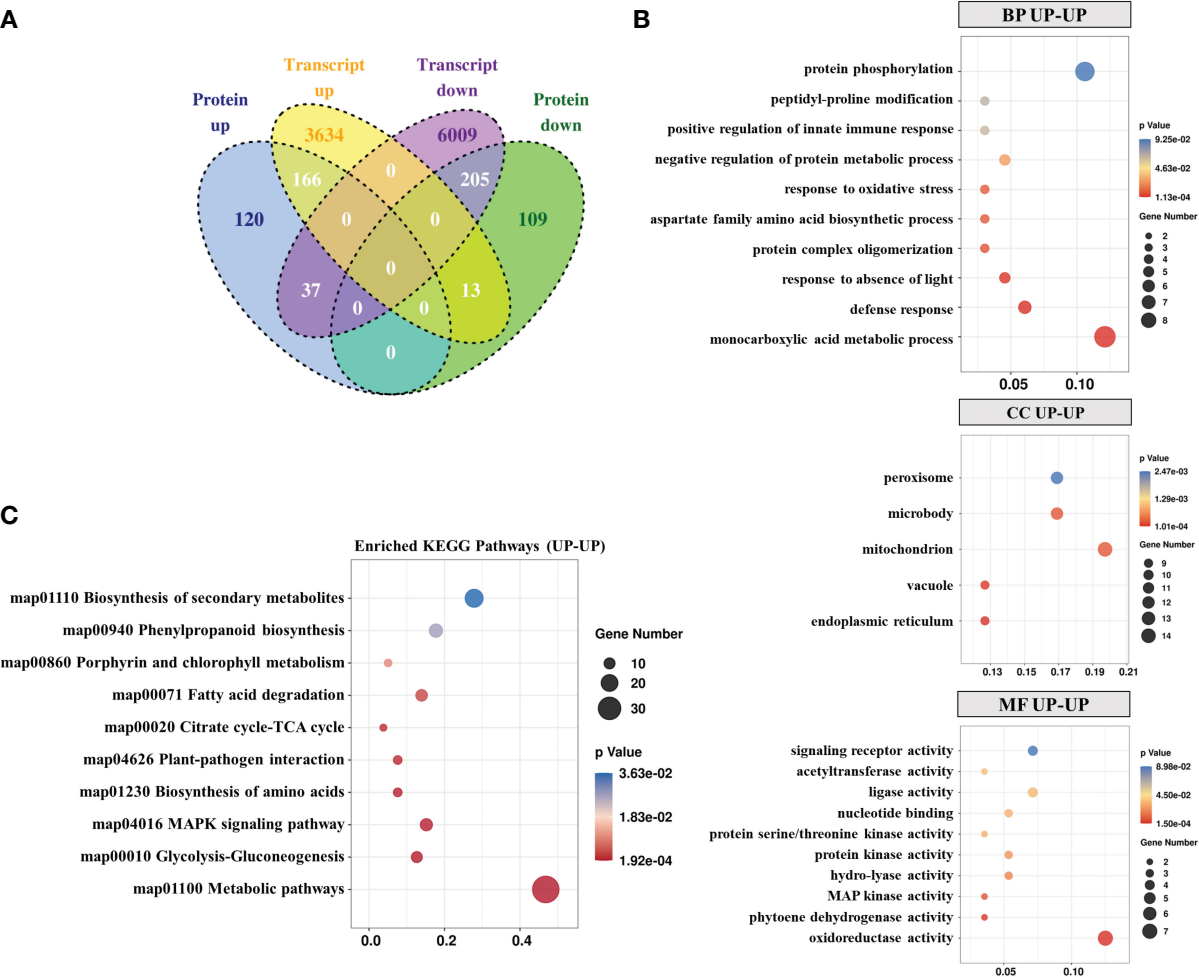
Integration of transcriptomic and proteomic data. **(A)** Venn diagram of the transcriptome and proteome. GO **(B)** and KEGG **(C)** analysis of upregulated DEPs-DEGs in the proteome and transcriptome.

To determine genes regulated at the protein and transcription levels, quantitative proteomic analysis was performed with transcriptomic data sets that used identical materials and inoculation methods. When the quantized protein matched the corresponding mRNA expression, a correlation between DEGs and DEPs was found. Overall, 166 out of 322 proteins were up-regulated at the transcription level and 205 out of 327 were down-regulated at the protein and transcription levels (**Figure 3A**). GO enrichment analysis revealed that most of the co-upregulated DEGs-DEPs were involved in “protein phosphorylation”, “monocarboxylic acid metabolic process” and “defense response” (**Figure 3B**). In the MF sub-categories, enzymatic activity related to MF (oxidoreductase, phytoene dehydrogenase, MAP kinase, hydro-lyase, and ligase activity) was significantly enhanced. Moreover, KEGG pathway analysis indicated significant enrichment of “Metabolic pathways”, “MAPK signaling pathway”, and “Plant-pathogen interaction”-related co-upregulation of DEGs-DEPs (**Figure 3C**).

### 3.4 Phosphorylation dynamics of rice variety MWG in response to *Magnaporthe oryzae* infection

**Figure 4:**
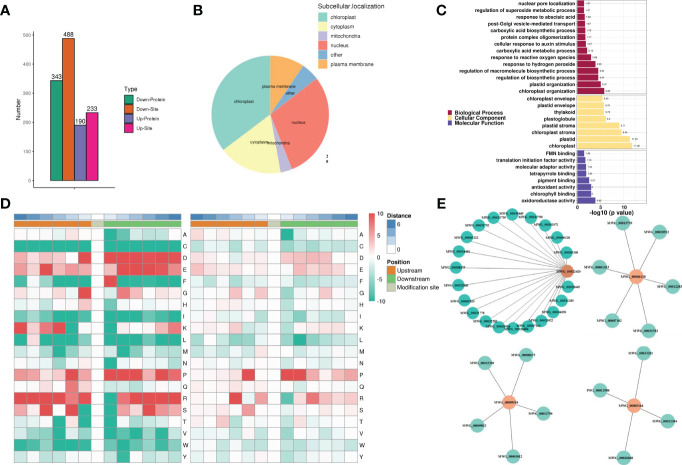
Phosphorylation dynamics of rice variety MWG in response to *Magnaporthe oryzae* infection. **(A)** Analysis of differentially modified sites. **(B)** The subcellular localization of phosphoproteins. **(C)** GO functional enrichment analysis of phosphoproteins. **(D)** Conserved amino acid around the modified serine residues and tyrosine residues from −6 to +6 positions. **(E)** Interaction network of phosphoproteins.

To profile phosphorylation events during rice variety MWG in response to *M. oryzae* infection, 5529 modified peptides and 1448 quantifiable proteins were identified, collectively containing 6345 identified phosphorylation sites (**Supplementary Table S2**). Among the identified phosphorylation sites, 5559 (87.61%) were from phosphoserine serine, 761 (11.99%) were found at threonine residues, and 25 (0.39%) were found at tyrosine residues. Most phosphopeptides were singly or doubly phosphorylated (**Supplementary Figure S4A-B**). The previously described DEP selection criteria were used to identify phosphoproteins with a differential abundance. A total of 533 differential abundance phosphoproteins were identified, of which 190 were up-regulated and 343 were down-regulated (**Figure 4A**). Given that phosphorylation can alter the subcellular localization of proteins, subcellular localization of proteins was found. Notably, the nucleus percentage exceeded the cytoplasm’s (**Figure 4B**).

Based on GO (**Figure 4C**) and KEGG (**Supplementary Figure S4C**) analyses, most of the protein phosphorylation events were related to several processes (antioxidant and oxidoreductase) and response (ROS and hydrogen peroxide). MF category illustrated the high accumulation of phosphoproteins involved in oxidoreductase and antioxidant activities and binding (chlorophyll, pigment, and tetrapyrrole). The motif-X elicitation (MEME) suite was used to analyze the amino acids surrounding the phosphorylated residues generated after *M. oryzae* infection to extract the conserved motifs of phosphopeptides. Ser and Thr phosphorylation contained nearly all the localized phosphosites, with a much higher frequency for phosphoserine motifs. A total of 49 phosphoserine motifs and 9 phosphothreonine motifs were identified (**Supplementary Table S3** and **Figure 4D**). The conserved motifs with the highest motif score of phosphopeptides were [S_PxR], [S_P], and [S_PK], which were the typical proline-directed motifs and the potential substrates of MAPK and S/TK. STRING analysis showed the possible protein-protein interaction (PPI) network among these phosphoproteins. MWG_00022420 (Oxygen-evolving enhancer protein 1, OEE1), MWG_00006128 (MAPK2), MWG_00009518 (Perox4), and MWG_00003164 (Serine/threonine-protein kinase, TOR1) were identified as hubs in the MWG in response to *M. oryzae* infection phosphoproteins-phosphoproteins interaction network, which connected with 21, 6, 5, and 4 nodes, respectively (**Figure 4E**).

### 3.5 Resistance of MWG against *Magnaporthe oryzae* and its physiological and biochemical mechanism

**Figure 5:**
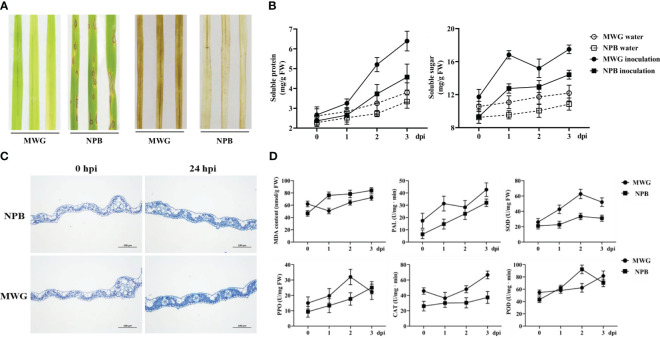
Resistance of rice variety MWG against *Magnaporthe oryzae* and its physiological and biochemical mechanism. **(A)** Blast symptoms and H_2_O_2_ accumulation of MWG and NPB. **(B)** The changes of soluble protein and sugar of MWG and NPB. **(C)** Callose accumulation of MWG and NPB. **(D)** The changes of MDA content and defense-related enzyme activities in MWG and NPB.

Comprehensive multi-omics analyses indicated that enzyme activity and redox-related genes/proteins are significantly induced in response to the infection with *M. oryzae* of MWG. Several physiological and biochemical indexes were investigated to determine the resistance mechanism of MWG. MWG and NPB were inoculated with *M. oryzae* to evaluate the resistance. The results showed that NPB is severely infected, with lesions spreading. However, MWG showed no symptoms (**Figure 5A**). In addition, a significant accumulation of H_2_O_2_, showing as brown staining, was detected in MWG leaves at 24 dpi. NPB only showed weak accumulation. MWG consistently had higher soluble sugar and protein content before and after inoculation with *M. oryzae* than NPB (**Figure 5B**). Furthermore, after inoculation with *M. oryzae*, callose deposition in MWG leaves was more than in NPB leaves (**Figure 5C**). Activities of antioxidant defense enzyme including CAT, PAL, PPO, POD, and SOD, and MDA content are shown in **Figure 5D**. After inoculation with *M. oryzae*, the PAL, PPO, SOD, and CAT activities in MWG were higher. Thus, the MWG blast resistance process was accompanied by a series of physio-biochemical changes such as ROS accumulation, callose deposition, soluble sugar and soluble protein content, and the activities of enzymes in antioxidant defense systems.

## 4 Discussion

High-throughput or multi-omics technologies have evolved in the last few decades in which single or multiple genes or proteins and the data-intensive and unbiased realm of transcriptomics or proteomics have been studied (Zhou et al., 2022). The mechanism of plant defense response against *M. oryzae* is complex. Once the pathogen attacks, plant metabolism has to balance the conflicting demands for resources to support the defense process rather than growth and development (He et al., 2022). Previous studies have identified a novel resistance gene with multiple disease resistance and stable yield from the elite hybrid rice restorer line R2115 and the blast-susceptible japonica accession TP309 through comparative transcriptomics. The findings revealed a broad-spectrum disease resistance mechanism that reduces plant immunosuppression through proteomics (Hu et al., 2023). This study used an integrated omics approach combining transcriptomic, proteomic, and phosphoproteomic analysis to investigate the internal dynamic functional changes involved in MWG in response to *M. oryzae* Infection. The integrative transcriptome and proteome analysis identified the protein biosynthetic process as differentially expressed GO terms. Several GO terms were related to disease resistance (NB-ARC domain, peroxidase, and glycosyl hydrolases family). Moreover, MWG consistently had a higher soluble protein content and activities of the pathogenesis‐related defensive enzymes PPO and PAL before and after inoculation with *M. oryzae* than NPB. These findings were consistent with the findings of a previous study that the protein content positively correlates with rice blast resistance, and resistance cultivars potentially induce the synthesis of more defense-related proteins (Yin et al., 2021). Sugars are the essential nutrient and effective signaling molecules in several plant life activities. The plant and the pathogen constantly struggle to utilize the host glucometabolic mechanisms, resulting in disease resistance or susceptible phenotype alteration (Kanwar and Jha 2019). Based on the integrative analysis, “Glyolysis-Gluconcogenesis” and “glucan biosynthetic and metabolic process” were significantly induced. This finding was possibly due to the invasion of pathogens-induced MWG that produces more sugars, ensuring adequate energy to synthesize more disease-resistant secondary metabolites (such as terpenoids, callose, and phenolics). Therefore, this phenomenon enhances MWG blast disease resistance. This phenomenon was also verified using physiological experiments (e.g., callose deposition, protein content, and sugar).

ROS participate in signal transduction and immune response in plants. The superoxide anion and hydrogen peroxide (H_2_O_2_) are the common ROS that directly inhibit or eliminate phytopathogens (Kang et al., 2021). In this study, DAB staining showed that MWG accumulated more ROS than NPB, which aligns with the pathway enrichment analysis of the transcriptome and proteome. Dysregulation of plant metabolism caused by biotic stress is frequently associated with ROS burst and oxidative stress, leading to nucleic acids and proteins dysfunction and irreversible damage to cell membrane lipids (Mohammadi et al., 2021). To prevent damage from oxidative stress, plants have evolved a robust antioxidant defense system to eliminate ROS. The antioxidative enzyme system is a critical component of plant immunity, and the alteration of its activity affects the degree of plant resistance (Zhang et al., 2022). The results and several physiological indices demonstrated that the antioxidative enzyme-related gene or protein is up-regulated at the transcriptional and translational levels. This finding corresponds with the results of omics studies on resistant cultivated rice, wild rice, and barley after *Rhizoctonia solani* infection (Shamim et al., 2022). Furthermore, *M. oryzae* infection increases ROS production in MWG, stimulating antioxidant enzymes to counteract oxidative damage and detoxify ROS.

Regarding integrative analysis, proteins strongly correlated with transcripts can be evaluated indirectly through transcriptomic analysis. However, transcriptomic analysis is inadequate for proteins not correlated with their transcripts. Based on this inconsistent and opposite expression trend of the gene mRNA and protein levels, it was speculated that the trend was because of different regulatory mechanisms at the transcriptional and translational levels: First, changes in mRNA expression at the transcript level may not always exactly and immediately be reflected in alterations at the protein level (Fang et al., 2015). Secondly, a complex series of signaling cascades are initiated through phytopathogens, resulting in an amplified signal. Finally, PTM may be responsible for this discrepancy.

In rice, protein phosphorylation is essential for the immune responses to phytopathogens infections (Hou et al., 2019). The difference and significance of phosphorylation in resistant and susceptible cultivars was further revealed based on the results of a previous phosphoproteome analysis (based 2-dimensional Electrophoresis) in rice (Li et al., 2015). In line with previous phosphoproteomics studies of rice exposed to bacterial blight, we found that many serine and threonine residues and few tyrosine residues were phosphorylated (Hou et al., 2015). Among them, the [S_P] motif exhibited the highest frequency and was generally considered to be a substrate of RLK, CDPK and MAPK (Hou et al., 2017; Wang Y. et al., 2017). Through a combination of transcriptomics, proteomics, and phosphoproteomics analysis, we found that the MAPK signaling was one of the highly activated pathways. The MAPK signaling cascade has been shown to play an important role in the regulation of responses to biotic stresses (Zhang et al., 2021; Wang et al., 2022). PPI network analysis of phosphorylated proteins indicated that OEE1, MAPK2 and Perox4 were the key hub proteins involved in MWG response to *M. oryzae* infection. By searching phosphorylation sites in this phosphoproteomics, we identify that two high-confidence predicted phosphorylation sites of MAPK2 were S511 and S515, and this needs to be further explored. Similarly, a comparative transcriptome studies of resistant rice cultivars Hui1586 and susceptible rice cultivars demonstrated that Perox4 was among the core genes that involved in the regulation of rice response to *M. oryzae* (Yang et al., 2021). In addition, two-dimensional polyacrylamide gel electrophoresis-based proteomics of the defense response of soybeans against *Bipolaris maydis* showed that OEE was tightly associated with occurrence of plant nonhost resistance (Dong et al., 2015). In tobacco, NbOEE2 interacts with the *Phytophthora capsici* effector RxLR19781 and the loss of NbOEE2 in tobacco alleviates resistance to *P. capsica* (Liang et al., 2019). Here, we observed that OEE1 was phosphorylated at 24 h after MWG response to *M. oryzae* infection, whereas its transcription and protein expression were downregulated. This is possibly because OEE1 plays a role in the early pathogen effector recognition reaction, suggesting that OEE1 expression analysis or functional verification should be performed at an earlier time point following pathogen infection in future studies. Although the precise function and mechanisms of candidate genes remain to be elucidated, our results provide a strong foundation and a technology platform for further investigations and functional analyses.

## 5 Conclusion

Rice blast is one of the most devastating diseases that restricts rice production. However, due to the variability of the rice blast fungus, resistance genes can easily lose their effectiveness. Therefore, exploring broad-spectrum disease-resistant breeding resources and analyzing the mechanism of disease resistance is crucial to combat this disease. In this study, we aimed to further understand the interaction between rice and the rice blast fungus. We analyzed the changes in the transcriptome, proteome, and phosphorylation modification of the broad-spectrum and persistent rice blast-resistant local variety MWG during the infection process of the rice blast pathogen. We also delved into the functional pathways and genes closely related to the MWG response to rice blast infection. Our transcriptomic analysis showed that rice blast infection caused significant transcriptional reprogramming in MWG, with the expression levels of many genes significantly altered. To confirm the reliability of the transcriptomic data, we conducted qPCR experiments. Additionally, our proteome and phosphoproteomic analysis found that rice blast infection caused significant changes in the phosphorylation modification level of MWG. Based on multi-omics data analysis, we found that MWG upregulated immune-related pathways in response to rice blast infection, including peroxide accumulation, oxidoreductase activity, and callus accumulation, among other plant defense pathways. We further measured multiple physiological and biochemical indicators in MWG before and after inoculation with the rice blast pathogen. In summary, our study sheds light on the molecular mechanism of rice blast development and has broader applications in designing new strategies for disease-resistant breeding.

## Data availability statement

The datasets presented in this study can be found in online repositories. The names of the repository/repositories and accession number(s) can be found in the article/**Supplementary Material.**

## Author contributions

WP: Performed the experiments and prepared draft of paper. YW and XZ: Data curation. WL and JL: Resources, Reviewing and editing. LD and BW: Conceived and designed the experiments, and funding acquisition. All authors contributed to the article and approved the submitted version.

## Supplementary material

Supplementary Figure 1 Principal component analysis of multi-omics.

Supplementary Figure 2 Alternative splicing events in the MWG transcriptome.

Supplementary Figure 3 Distribution of peptide length and protein molecular weight.

Supplementary Figure 4 Characteristics of MWG phosphoproteomics. **(A)** The type of phosphorylation modification sites. **(B)** The number of phosphorylation modification sites. **(C)** The subcellular localization of phosphoproteins.
