# Supplementary figures and images for: Integrative transcriptomic, proteomic, and phosphoproteomic analysis on the defense response to Magnaporthe oryzae reveals different expression patterns at the molecular level of durably resistant rice cultivar Mowanggu
Source: Front Plant Sci. 2023 Jul 13;14:1212510. doi: 10.3389/fpls.2023.1212510 (PMC10373791; doi:10.3389/fpls.2023.1212510)

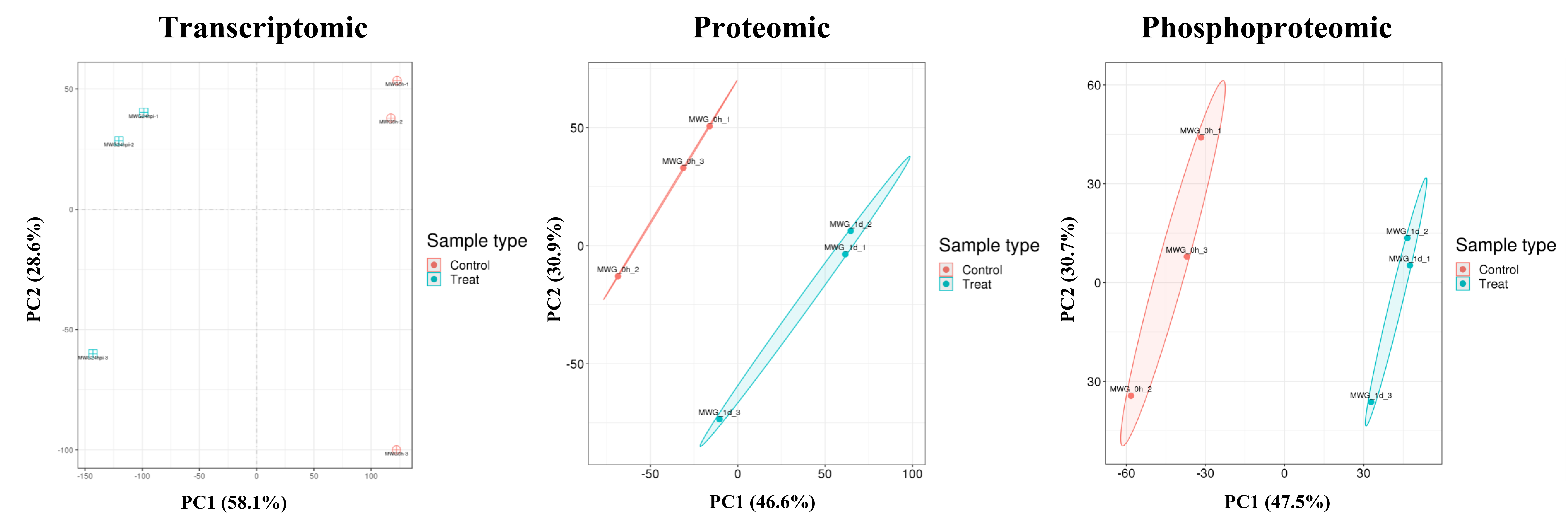

Supplement: Supplementary Figure 1 — Principal component analysis of multi-omics. [file Image_1.jpeg]

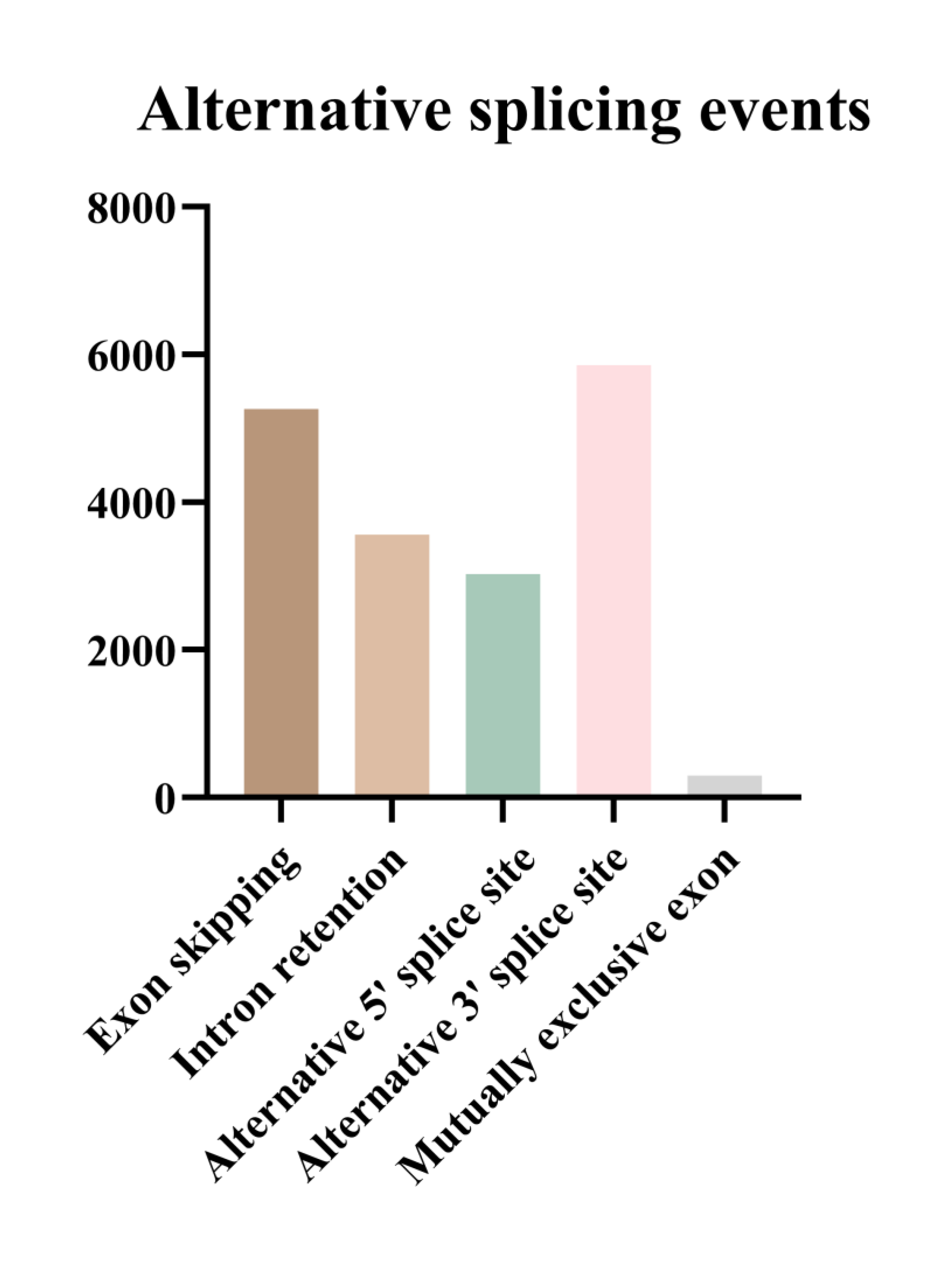

Supplement: Supplementary Figure 2 — Alternative splicing events in the MWG transcriptome. [file Image_2.jpeg]

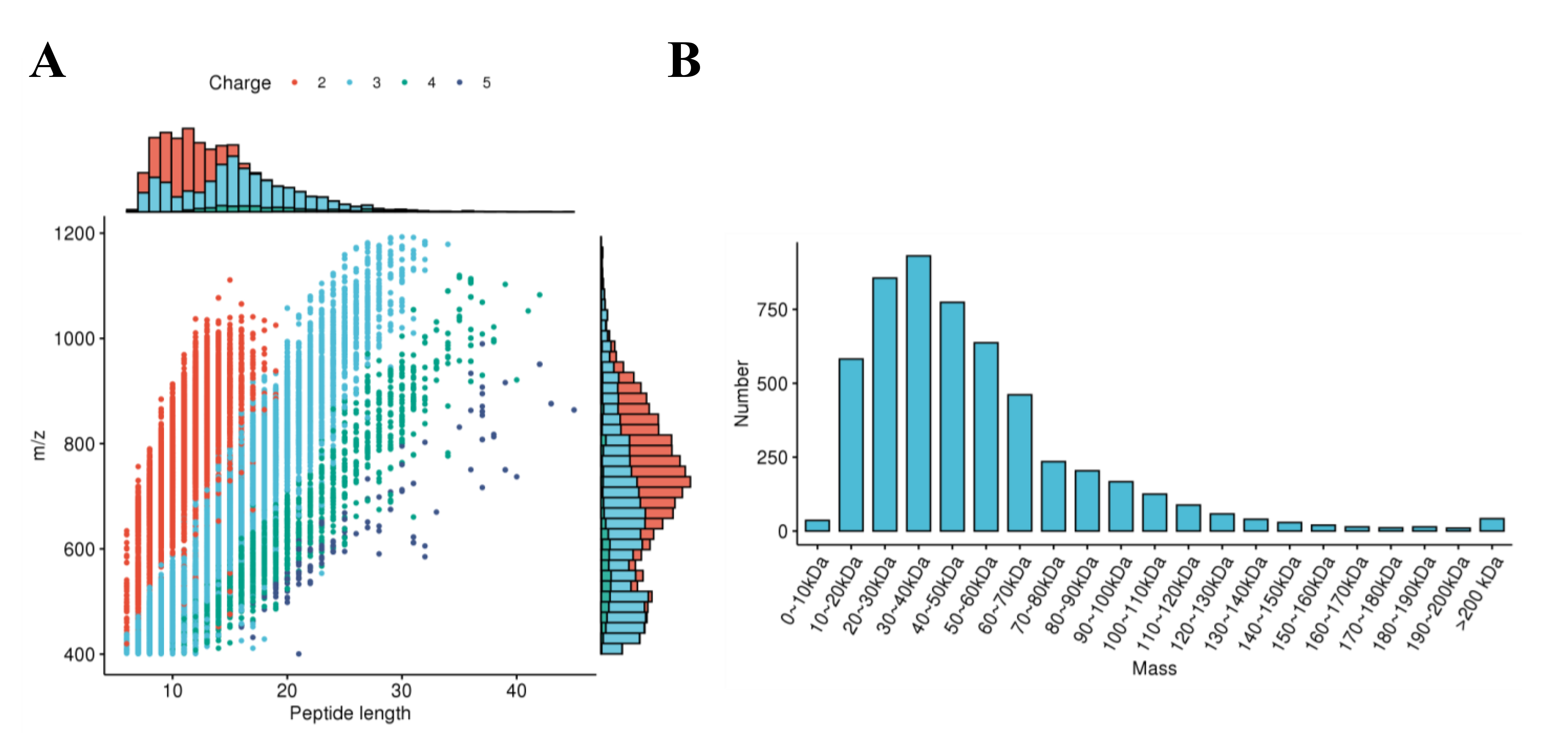

Supplement: Supplementary Figure 3 — Distribution of peptide length and protein molecular weight. [file Image_3.jpeg]

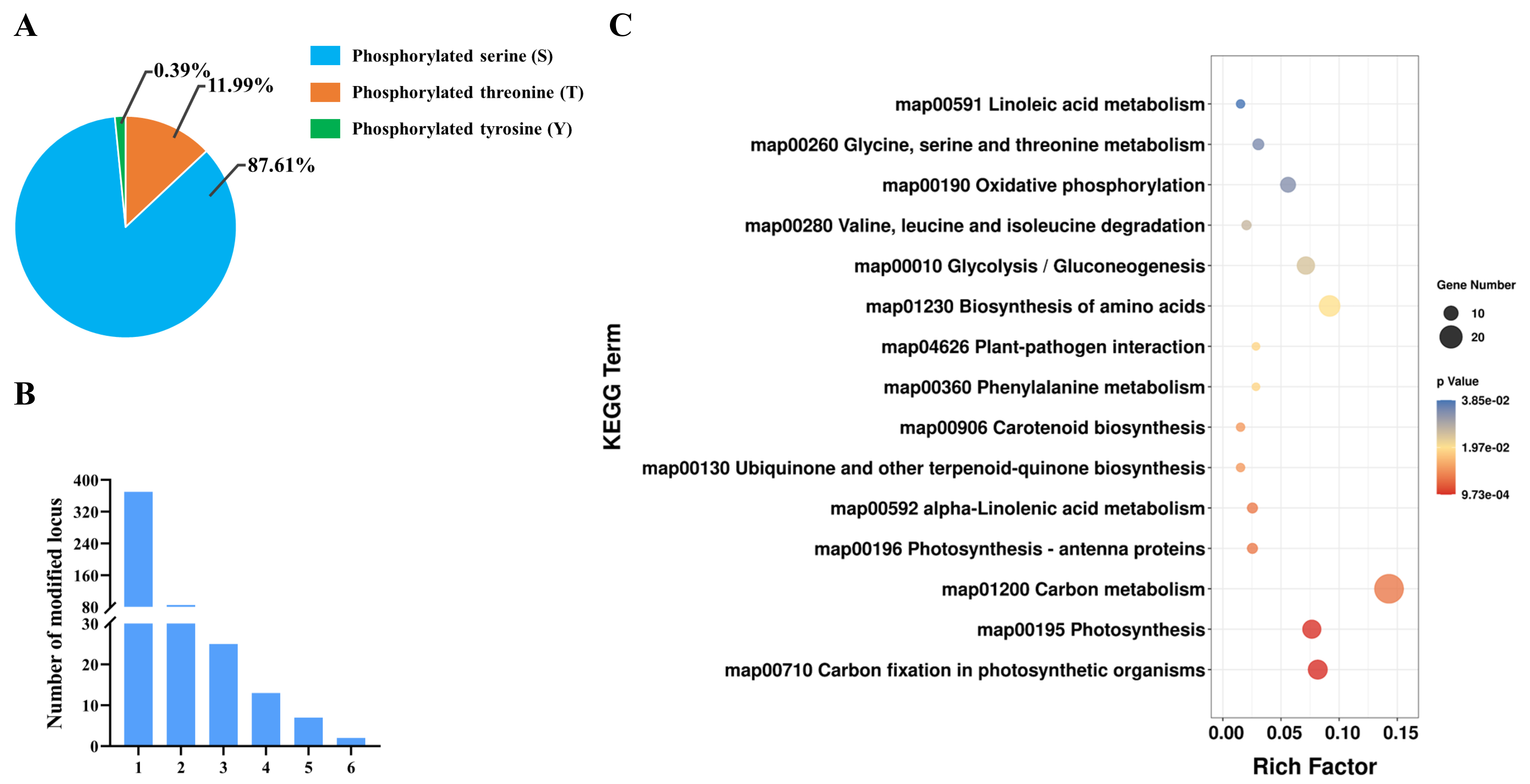

Supplement: Supplementary Figure 4 — Characteristics of MWG phosphoproteomics. (A) The type of phosphorylation modification sites. (B) The number of phosphorylation modification sites. (C) The subcellular localization of phosphoproteins. [file Image_4.jpeg]
